# Transcriptomic characterization of *Coccidioides* morphological states using RiboMarker-enhanced RNA sequencing

**DOI:** 10.1093/g3journal/jkag111

**Published:** 2026-04-28

**Authors:** Jonathan M Howard, Aidan C Manning, Rachel C Clark, Tahirah Williams, Clarissa J Nobile, Sergei A Kazakov, Sergio Barberan-Soler

**Affiliations:** RealSeq Biosciences, Santa Cruz 95060, United States; RealSeq Biosciences, Santa Cruz 95060, United States; RealSeq Biosciences, Santa Cruz 95060, United States; Department of Molecular and Cell Biology, University of California Merced, Merced 95343, United States; Quantitative and Systems Biology Graduate Program, University of California Merced, Merced 95343, United States; Health Sciences Research Institute, University of California Merced, Merced 95343, United States; Department of Molecular and Cell Biology, University of California Merced, Merced 95343, United States; Health Sciences Research Institute, University of California Merced, Merced 95343, United States; Center for Cellular and Biomolecular Machines, University of California Merced, Merced 95343, United States; RealSeq Biosciences, Santa Cruz 95060, United States; RealSeq Biosciences, Santa Cruz 95060, United States

**Keywords:** Valley fever, *Coccidioides*, fungal infection, RNA fragmentomics, RiboMarker, RNA sequencing, small RNAs

## Abstract

*Coccidioides* is a dimorphic fungal pathogen responsible for the mammalian disease commonly known as Valley fever. While previous studies have characterized transcriptomic changes associated with its life stage transitions, the contribution of small RNAs (sRNAs)—key regulators of virulence in other pathogenic fungi—remains unexplored. Here, we profile sRNA expression across three *Coccidioides posadasii* morphologies—arthroconidia, mycelia, and spherules—analyzing both intracellular and extracellular RNA fractions. Utilizing RiboMarker sRNA and RNA fragment library preparation, we achieved enhanced transcriptome coverage by incorporating RNA species that are typically underrepresented or incompatible with standard sequencing workflows. We observed pronounced transcriptomic remodeling during the transition from arthroconidia to mycelia or spherules, driven primarily by changes in protein-coding transcripts, tRNA, and unannotated loci. These data identify sRNA- and fragment-producing loci that may be required for progression between the saprobic and parasitic life cycles of *Coccidioides*. In addition, distinct RNA fragmentation patterns were associated with each morphological state. Notably, we detected evidence of RNA export to the extracellular space, particularly snRNAs and tRNA-derived fragments, during these transitions, suggesting potential roles in cell–cell or host–pathogen communication. Together, this intra- and extracellular *Coccidioides* sRNA atlas provides a resource for biomarker discovery and advances our understanding of the molecular basis of fungal virulence.

## Introduction

*Coccidioides*, which includes *Coccidioides immitis* and *Coccidioides posadasii*, is a genus of dimorphic fungal pathogens endemic to the arid and semi-arid regions of the southwestern United States and Mexico. Together, these species cause coccidioidomycosis, a disease commonly known as Valley fever (Nguyen et al. 2013). Recent estimates indicate that *Coccidioides* is responsible for nearly 20,000 clinically significant cases of Valley fever annually in the United States (Facts and Stats about Valley Fever. U.S. Center for Disease Control and Prevention: 2024), although this number is likely underestimated due to misdiagnosis, underreporting, and limited access to medical care (Galgiani et al. 2005). The incidence of Valley fever is projected to increase across much of the western United States, driven by multiple converging factors, including the emergence of antifungal-resistant fungal pathogens (Lockhart et al. 2023), a growing immunocompromised population (Odio et al. 2017; Martinson and Lapham 2024), and environmental changes associated with climate change (Williams and Chiller 2022). Consequently, the economic burden of coccidioidomycosis is expected to rise from an estimated $3.9 billion in 2015 to over $18 billion by the end of the 21st century (Gorris et al. 2021). Reflecting this growing public health concern, the World Health Organization included *Coccidioides* in its inaugural 2023 list of Fungal Priority Pathogens, highlighting the urgent need for expanded basic research and improved diagnostic and therapeutic tools (World Health Organization 2022).

To address these challenges, recent studies have used next-generation sequencing (NGS) approaches to characterize transcriptional programs underlying the saprobic-to-parasitic phase transition of *Coccidioides* (Carlin et al. 2021; Homer et al. 2025). RNA sequencing experiments comparing mutant strains and wild-type strains of *C. posadasii* have shown that differentiation into the parasitic spherule form is accompanied by increased expression of genes associated with nutrient acquisition (such as specific amino acid and sugar transporters), which are required for survival and proliferation within the unique, nutrient-restricted environment of the host (Narra et al. 2016). Similarly, profiling nascent transcriptomes across life cycle stages of *C. immitis* revealed differential expression of genes involved in stress responses, cell wall remodeling, polarized growth, transcriptional regulation, and known virulence pathways (Duttke et al. 2022). Many of these changes are linked to *cis*-regulatory elements bound by Ryp1, a conserved transcription factor that plays a central role in fungal developmental transitions (Mandel et al. 2022). More recently, *RYP1*-dependent arthroconidia-associated transcripts were shown to contribute directly to arthroconidial cell wall biology (Homer et al. 2025). Collectively, these studies have established a foundational understanding of protein-coding transcriptional networks that govern morphological transitions in *Coccidioides* and have identified potential targets for diagnostic and therapeutic intervention.

Despite these advances, existing transcriptomic analyses largely overlook or actively select against small noncoding (sRNAs) and RNA fragments. Consequently, the specific contributions of sRNAs to the morphological plasticity and virulence of *Coccidioides* remain entirely uncharacterized, representing a critical knowledge gap in our understanding of Valley fever pathogenesis. sRNAs are broadly defined as RNA molecules shorter than 200 nucleotides derived from diverse subclasses, including tRNAs, miRNAs, snRNAs, and snoRNAs, that perform a wide range of biological functions (Gou et al. 2023). This category also includes RNA fragments generated through regulated or incidental endo- and exonucleolytic processing, such as tRNA-derived fragments (tRFs/tDRs) and rRNA-derived fragments (rRFs), which may function as regulatory RNAs or represent intermediates of RNA processing and decay (Zinder and Lima 2017; Lambert et al. 2019; Chen and Zhou 2023). In eukaryotic pathogens, including fungi and oomycetes, sRNAs play central roles in regulating virulence and host–pathogen interactions. For example, the oomycete pathogen *Phytophthora sojae* produces sRNAs that mediate transgenerational silencing of the virulence gene *Avr3a*, thereby enhancing pathogenicity and immune evasion in subsequent generations (Qutob et al. 2013). Similarly, in the soilborne fungal pathogen *Verticillium dahliae*, the microRNA-like RNA VdmilR1 represses the essential pathogenicity gene *VdHy* through chromatin-mediated silencing mechanisms (Jin et al. 2019). Together, these findings underscore the importance of sRNAs as regulators of dynamic gene expression programs across pathogen life cycles and during infection.

In addition to their intracellular roles, sRNAs are increasingly recognized as components of extracellular vesicles (EVs) released by fungal pathogens. EV biogenesis and trafficking facilitate the targeted delivery of fungal macromolecules—including proteins, lipids, and RNAs—to host cells and tissues, contributing to infection and immune modulation (Lai et al. 2023b). Cross-kingdom RNA interference mediated by EV-associated miRNAs, milRNAs, and siRNAs has been documented in several fungal–host systems (Katiyar-Agarwal and Jin 2010; Islam et al. 2017; Hua et al. 2018). For instance, *Botrytis cinerea* exports small RNAs into plant host cells, where they hijack host silencing machinery to suppress immune-related gene expression (He et al. 2023). Similarly, *Beauveria bassiana* delivers the milRNA bba-milR1 into mosquito hosts to inhibit immune signaling and promote infection (Cui et al. 2019). With these data, the transport and release of miRNAs/milRNAs into nearby host tissues have been shown to contribute, in part, to the pathophysiological regulation of fungal infections, most notably being cross-kingdom RNA interference (Cheng et al. 2023; Mahanty et al. 2023). However, many subtypes of sRNAs (e.g. snoRNAs, tRNAs/tRFs, snRNAs) are found to populate fungal EVs, many with canonical functions outside of the direct suppression of mRNA translation (Bitencourt et al. 2022). Identifying what RNAs are packaged in these EVs of *Coccidioides* is not only a first step toward uncovering their putative roles in host–pathogen interactions but is also an additional layer informing the discovery of RNA biomarkers (especially those found outside the cell) that could be prognostic of Valley fever infection.

To better characterize the small RNA profile of *Coccidioides*, we performed small RNA sequencing using an enhanced RealSeq-RiboMarker protocol to generate a high-resolution small RNA atlas of *C. posadasii*. We profiled across a 96-h growth time course of cultures for the saprobic mycelia and arthroconidia as well as from parasitic spherules. Additionally, we implemented the RealSeq RiboMarker method to reveal additional layers of full-length and fragment sRNAs often unincorporated into sequencing libraries. Furthermore, we interrogated cell-free and exosomal RNA from conditioned media for these morphologies to determine what, if any, small RNAs may be exported from the fungus into the extracellular space. Our data reveal that morphological changes are not only driven by protein-coding genes, but also by annotated sRNA types, including tRNA fragments (tRFs/tDRs), as well as unannotated sRNA loci. Additionally, we identified the export of key EV-associated RNAs which may define and shape cell:cell and pathogen:host interactions. Ultimately, this work strives to further characterize the distinct RNA profiles associated with these important life stage morphologies of *Coccidioides* and provide a foundational resource of potential RNA biomarkers to target in ex vivo samples of infected hosts.

## Materials and methods

### Culture conditions and harvesting

C. *posadasii* (strain Δ*cts*2/Δ*ard1*/Δ*cts3*, NR-166; BEI Resources) arthroconidia were harvested from 6-week-old plates as previously described (Mead et al. 2020). The spores were inoculated at 1 × 106 arthroconidia into a 250 mL baffled Erlenmeyer flask containing 100 mL of 2× GYE, shaking at 150 rpm at 30 °C for 48 h. Conditioned media was harvested by collecting media with cells into centrifuge tubes, which were centrifuged at 12,000 × *g* for 8 min to pellet the cells. The supernatant was pooled and filtered with a 0.22-µm syringe filter before being stored at −80 °C.

To generate samples for the time course, in which arthroconidia transition to mycelia, 1 × 10^6^ of harvested arthroconidia were incubated in 250 mL flat-bottom Erlenmeyer flasks (Corning, Corning, New York, United States) in 100 mL of 2× GYE media, shaking at 150 rpm at 30 °C. After 48 h, the conditioned media with cells (predominantly in the arthroconidia morphology) were centrifuged at 12,000 × *g* for 8 min to pellet the cells. Half of the filtered fungal cells were kept for RNA isolation, while the condition media was spun down, and supernatant was removed for extracellular RNA isolation. The remaining cells were reinoculated into fresh 2× GYE for an additional 24 h, shaking at 150 rpm at 30 °C. At this 72-h time point, the fungal cells (containing a mixture of both arthroconidia and mycelia morphologies) were separated from the growth media by filtering with a sterile 40 µm nylon mesh cell strainer. Half of the filtered fungal cells were kept for RNA isolation, while the conditioned media was filtered with a 0.22-µm syringe filter before being stored at −80 °C for extracellular RNA isolation. The remaining half of the filtered fungal cells was reintroduced again into fresh 2× GYE for 24 h, shaking at 150 rpm at 30 °C. This sample processing was performed again for the cell samples at the 96-h time point (predominantly in the mycelia morphology) for a total of 3 replicates of time course fungal cell samples and filtered conditioned media. *C. posadasii* spherules were propagated and harvested from 6-week-old plates as previously described (Mead et al. 2020). For spherule samples, 50 mL of Converse medium (Converse and Besemer 1959) was inoculated to a final concentration of 10^6^ arthroconidia/mL. Cultures were then incubated at 39 °C in 10% CO_2_, shaking at 150 rpm for ∼ 5 d. Spherules were harvested by filtering the culture through a nested filter into a 50 mL conical tube and were then centrifuged at 9000*×g* for 8 min to pellet the cells. Cell pellets were washed twice with 1× PBS and then snap frozen for storage at −80 °C until use.

### Fungal and extracellular RNA extraction and purification

C. *posadasii* mycelia, arthroconidia, and spherules samples were stored in RNA Shield (Zymo Research) at −80 °C until processing using the Quick-RNA Fungal/Bacterial Microprep kit (Zymo Research) according to the manufacturer's instructions. Samples were added to a pre-chilled 2 mL ZR Bashing Bead lysis tube with 0.5 mm beads (Zymo Research), and tubes were arranged in a FastPrep-24 grinder and lysis system (MP Biomedicals) and disrupted 5× for 60 s at 6.5 m/s, with intervening incubations on ice for 5 min. DNase I treatment on-column was also implemented. Cell-free and exosome RNAs from *C. posadasii* mycelia and arthroconidia samples were isolated from 40 mL of conditioned culture media using the Plasma/Serum Exosome and Free-Circulating RNA Isolation Kit (Norgen Biotek) according to the manufacturer's instructions. Eluted total and extracellular RNAs purified from mycelia, arthroconidia, and spherules samples (*n* = 1 biological replicate per morphological stage for extracellular fractions), as well as conditioned media, were quantified using a Qubit 3.0 fluorometer (Invitrogen).

### RNA pretreatment with RiboMarker

For treatment of RNA with the RiboMarker platform, 10 µL of RNA sample was incubated in a 15 μL reaction mixture containing 1.5 μL RiboMarker Buffer 1, 1.5 μL of RiboMarker Enzyme 1, and 2 μL of RNase-Free H2O at 37 °C for 10 min, followed by 80 °C for 2 min. Next, 2 μL of RiboMarker Buffer 2 and 2 μL of RiboMarker Enzyme 2 were added to the mixture, which was then incubated at 37 °C for 60 min. Finally, 1 μL of RiboMarker Buffer 3 was added to the reaction and incubated at 37 °C for 60 min. The pretreated RNA was isolated using an RNA Clean and Concentrator-5 kit (Zymo Research) for downstream use in library preparation.

### Library preparation and sequencing

For small RNA-seq, libraries were prepared from total RNA using the RealSeq-Biofluids library preparation kit (RealSeq Biosciences) according to the manufacturer's instructions. Libraries for each ecotype, as well as cell-free and exosome samples, were generated separately and then pooled for the analysis. While arthroconidia and spherule intracellular samples were sequenced in triplicate, 1 mycelium biological replicate failed initial library preparation quality control. Thus, only 2 biological replicates for the mycelium morphology were utilized in downstream analyses. It should be noted that due to the limited number of biological replicates (*n* = 2 or 3 for intracellular datasets), our statistical power to detect small-magnitude differential expression is constrained. Consequently, transcripts or loci designated as not significantly differentially expressed may include false negatives and should be interpreted with appropriate caution. Finally, prepared libraries were pooled and subjected to single-end 75 cycles of sequencing using the F3 50 cycle sequencing kit (note: reagents for 50 additional cycles above what is represented were included to account for index sequencing needs) on the G4 system according to the manufacturer's instructions (Singular Genomics).

### Data analysis

Sequencing reads were trimmed of adapter sequence using cutadapt version 4.6 [–nextseq-trim = 15 -u 1 -a TGGAATTCTCGGGTGCCAAGG -m 15] (Martin 2011). Reads were then aligned to the *C. posadasii* strain Silveira (GCA_000170175.2) with the addition of the high-confidence set of tRNA isodecoders containing -CCA tails identified using tRNAScan-SE (Chan et al. 2021). ShortStack v4.1.0 (Axtell 2013) with the parameters [-pad 3 –mincov 0.5 rpm] was used to initially identify unannotated sRNA-producing loci. To strictly define these as potential novel sRNA transcripts, loci identified by ShortStack were subsequently cross-referenced against known structural and noncoding RNA databases (including RNACentral and Rfam) to filter out unannotated fragments of known conserved house-keeping RNAs. Loci lacking homology to known databases, yet exhibiting morphology-specific expression patterns, were retained as novel candidates. Read counts were normalized using DESeq2 version 1.42.0 (Love et al. 2014).

### RNA fragmentation analysis

Read pileups across the body of noncoding RNA (ncRNA) transcripts, from 5′ to 3′, were extracted and converted into a linear vector representing read coverage across each molecule. Coverage was normalized to the total number of reads mapping to each transcript, and a 1-way functional ANOVA was applied to assess significant differences in coverage patterns across morphologies, using a threshold of *P* < 0.05. To further explore these patterns, we employed a probabilistic functional data clustering approach, specifically fuzzy k-means, to classify transcripts based on their normalized read coverage profiles. The model was trained with 3 clusters corresponding to the distinct *Coccidioides* life morphologies: arthroconidia, mycelia, and spherule. Each sample was subsequently assigned probabilities reflecting the strength of association between its coverage pattern and the representative functional cluster for each morphology. For each transcript, a “cluster_score” was calculated that reflected the strength of the fuzzy k-means approach for identifying morphology-specific fragmentation patterns with a score of 1 indicating perfect morphology-driven patterns and <1 indicating samples from different morphological stages had similar fragmentation patterns.

## Results

### RiboMarker-enhanced RNA sequencing of *Coccidioides* morphologies

Most conventional small RNA (sRNA)-Seq library preparation kits are limited to the capture of RNA species with 5′-phosphate, 3′-hydroxyl, and minimal (if any) internal base modifications. Recent data identifies only a small portion (∼10%) of any small RNA transcriptome may be endogenously compatible (Lai et al. 2023a), suggesting many of these RNA species are not adequately captured using commercially available library preparation kits. To address these limitations, we investigated potential improvements to pretreatments collectively referred to as “end-healing,” or the chemical/enzymatic conversion of incompatible RNA ends to those required for adapter ligation during library preparation. T4 Polynucleotide Kinase (T4PNK) is a commonly used enzyme to achieve compatible ends due to its dual 5′ kinase and 3′ phosphatase activities (Novogrodsky and Hurwitz 1966; Cameron and Uhlenbeck 1977); however, the optimum conditions for PNK reactions have not been determined when detecting naturally occurring RNA fragments.

**Fig. 1.**
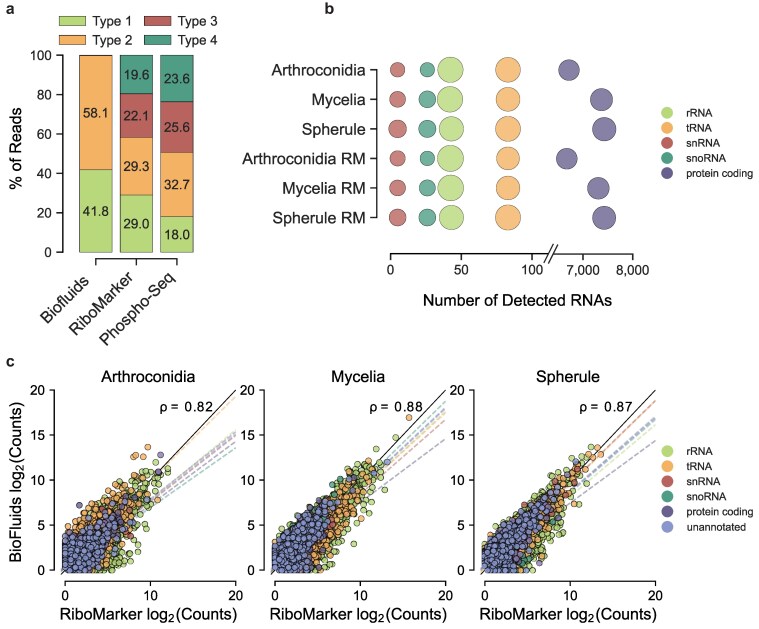
RNA sequencing across 3 *Coccidioides* morphologies of *C. posadasii.* a) Bar plot showing percentages of spike-ins representing various RNA end types detected in libraries prepared with different methods: RealSeq-Biofluids Library Prep without RNA pretreatment (“Biofluids”), RealSeq -Biofluids Library Prep with T4PNK RNA pretreatment in RiboMarker Buffer (“RiboMarker”), and NEBNext Small RNA Library Prep with standard PNK pretreatment (“Phospho-Seq”). For Biofluids, type 3 and 4 reads are also below 1% and are thus not visible. Types are defined as follows: type 1 = 5′ phosphate, 3′ hydroxyl; type 2 = 5′ hydroxyl, 3′ hydroxyl; type 3 = 5′ hydroxyl, 3′ phosphate; type 4 = 5′ phosphate, 3′ phosphate. b) Dot plot representing the number of detected transcript annotations mapped to *C. posadasii* in Biofluids (top) and RiboMarker (bottom) libraries, with dot size proportional to the abundance of each RNA class. c) Scatter plot of the abundance of non-redundant transcripts between Biofluids and RiboMarker for an arthroconidia (left; Pearson = 0.82), mycelia (middle; Pearson = 0.88), and spherules (right; Pearson = 0.87).

To interrogate the capacity of T4PNK to increase RNA inclusion in sequencing libraries, we tested different T4PNK pretreatments under conditions previously described (Cameron and Uhlenbeck 1977), to determine their respective effects on the sequencing output. We assessed 4 different buffers, the RiboMarker Buffer (RealSeq Biosciences), imidazole, TRIS in the pH range from 6.0 to 7.0, and the standard (NEB formulated) T4PNK Buffer (pH 7.6) supplied with the enzyme (as indicated in Supplementary Fig. 1), and performed experiments in the presence and absence of 10 µM ATP (or 1 mM ATP for standard conditions as suggested by the manufacturer), as sustained T4PNK activity may require the presence of this substrate (Cameron and Uhlenbeck 1977; Cameron et al. 1978). To establish a standardized measure of RNA diversity, we quantified the number of unique RNA transcripts detected per sample. To ensure comparability across libraries, reads were subsampled to a depth of 5 million, and a stringent detection threshold was applied, requiring at least 10 mapped reads per transcript. Using this metric, we observed statistically significant shifts (*P* < 0.05; Supplementary Fig. 1) in the diversity of captured RNA classes across PNK reaction conditions (“None” vs all treatments). However, T4PNK treatment in the presence of RiboMarker Buffer resulted in 2 of the most notable differences compared to other conditions. We observed a distinguishable increase in protein-coding gene-derived RNAs using RiboMarker conditions (Supplementary Fig. 1; “RiboMarker Buffer”), as well as a slight, but detectable increase in all other classes, apart from rRNAs (ribosomal) and scaRNAs (small Cajal-body-specific RNAs). To further validate the ability of RiboMarker Buffer to increase RNA inclusivity, we sequenced a pool of synthetic spike-ins (see Supplementary File 1) alongside these natural RNAs, which contained all potential end type combinations (type 1 = 5′ phosphate, 3′ hydroxyl; type 2 = 5′ hydroxyl, 3′ hydroxyl; type 3 = 5′ hydroxyl, 3′ phosphate; type 4 = 5′ phosphate, 3′ phosphate). Libraries were prepared using the RealSeq-Biofluids kit, both without RNA pretreatment (“BioFluids”) and with T4PNK treatment in RiboMarker Buffer (“RiboMarker”), and a library using an alternative small RNA library preparation kit with standard T4PNK treatment in the presence of ATP (“Phospho-Seq”) (Giraldez et al. 2019). These results (Fig. 1a) confirmed that T4PNK treatment in RiboMarker Buffer resulted in the inclusion of a more diverse set of RNA molecules containing both compatible and previously incompatible RNA ends into the sequencing library and yielded a more evenly balanced read distribution for all 4 RNA end types vs the alternative kit with standard T4PNK treatment. Therefore, these data revealed that T4PNK RNA pretreatments, in the presence of RiboMarker Buffer, facilitated the most unbiased, and therefore diverse, inclusion of RNA molecules into resulting libraries.

To expand upon the use of RiboMarker into a new sequencing space, we chose to characterize the dynamic changes in small RNA expression across the multiple morphologies of *Coccidioides*, an epidemiologically important, yet understudied, fungal pathogen (Kollath et al. 2019). We utilized *C. posadasii*, Δ*cts2*/Δ*ard1*/Δ*cts3*, NR-166 as our model strain (Xue et al. 2009), and harvested arthroconidia, mycelia, and spherules samples, representing several key morphologies for this important fungal pathogen. Arthroconidia were inoculated into growth media and grown for 4 d, with time points harvested at 48 h (arthroconidia) and 96 h (mycelia), representing the transition of *Coccidioides* arthroconidia to mycelia. Additionally, to generate spherules, the same arthroconidia were harvested after growing for 5 d in Converse media (Converse and Besemer 1959). For assessing the presence of potential extracellular *Coccidioides* RNAs, conditioned growth media from all morphologies were also collected and used for cell-free RNA (cfRNA) and exosomal RNA isolation. Additionally, each sample was subjected to 2 different RNA pretreatment conditions: one utilizing no RNA pretreatment and one implementing the optimized T4PNK pretreatment in RiboMarker Buffer (Fig. 1a). Together, these 2 datasets enabled us to comprehensively evaluate the small RNA transcriptome of each morphology tested as well as ascertain what differences, if any, in RNA expression and fragmentation occurred across the saprobic and parasitic life cycles of *Coccidioides*.

The resulting sequencing libraries were processed (see Materials and methods), and the differences across the morphologies of *C. posadasii* were explored using both Biofluids and RiboMarker library preparations. An initial assessment of the read lengths suggested that RiboMarker reduces biases to specific RNA types (Supplementary Fig. 2). For example, the arthroconidia samples (Supplementary Fig. 2a; “Arthroconidia”) exhibited large peaks at ∼ 28 nt, often indicative of the inclusion of specific RNAs, while a more even distribution from ∼15 to 35 nt was observed with RiboMarker pretreatment. Read length patterns for intracellular spherules (Supplementary Fig. 2a; “Spherule”) looks similar between “BioFluids” and “RiboMarker,” apart from a more pronounced peak at ∼35 nt, possibly indicative of tRNA halves or fragments (Fu et al. 2023). Looking at the pool of distinct RNA molecules captured from each morphology in our panel using both Biofluids and RiboMarker T4PNK pretreatment (Fig. 1b and c), we found a strong correlation between these library preparations (Fig. 1c). However, these data also point to the enrichment of key sRNA sub-populations in the RiboMarker samples that may otherwise not be detected using no treatment approaches (Fig. 1c; hatch marks). Indeed, we noted large increases in reads derived from ribosomal RNA (rRNA) across the arthroconidia and mycelia samples, as well as tRNA-derived reads in the spherules samples (Supplementary Fig. 3a), suggesting that RiboMarker treatment led to a shift in the incorporation of previously incompatible RNA ends that would otherwise be biased against (Hernandez et al. 2023). An assessment of the overlapping molecules captured using either Biofluids or RiboMarker, (Supplementary Fig. 4) revealed ∼50% to 60% RNAs were incorporated using either method, while the remaining ∼40% to 50% of reads showed specific inclusion in either Biofluids (∼13% to 16%) or RiboMarker (∼24% to 33% of all unique reads) preparations. By and large, we observe minimal differences in the detected expression across most annotated sRNA gene types (Fig. 1b), regardless of RNA pretreatment. However, we did observe differences in the number of reads mapping to *Coccidioides* protein-coding genes, especially in the spherules samples, suggesting that these sRNAs may be specifically upregulated, or are a byproduct of degradation/processing of larger mRNA species turned on during transition to this morphology (Carlin et al. 2021). Using a principal component analysis (Supplementary Fig. 5), we observe that regardless of the library preparation method, clear separation of each time point suggested distinct populations of small RNAs/RNA fragments were captured in the different *Coccidioides* morphologies. Taken together, these data suggest there are sub-populations of RNA that are more efficiently captured using RiboMarker, resulting in a more diverse, and unbiased, sequencing library. Furthermore, these results highlight that to completely capture the diversity of small RNA molecules found in each biological sample, multiple approaches may be needed, and using either approach, we note distinct small RNA profiles across *Coccidioides* life stages.

### Characterization of *Coccidioides* sRNA profiles from distinct morphologies reveals ecotype-specific differences in expression

While the transition of arthroconidia to mycelia or spherules is largely dependent on environmental factors, such as changes in CO_2_ levels (Klotz et al. 1984), recent literature has begun to tease apart the transcriptional reprogramming required to support the external cues that trigger transitions between *Coccidioides* morphologies (Carlin et al. 2021). We sought to further our understanding of the transcriptional changes driving *C. posadasii* morphological transitions using RiboMarker small RNA sequencing.

**Fig. 2.**
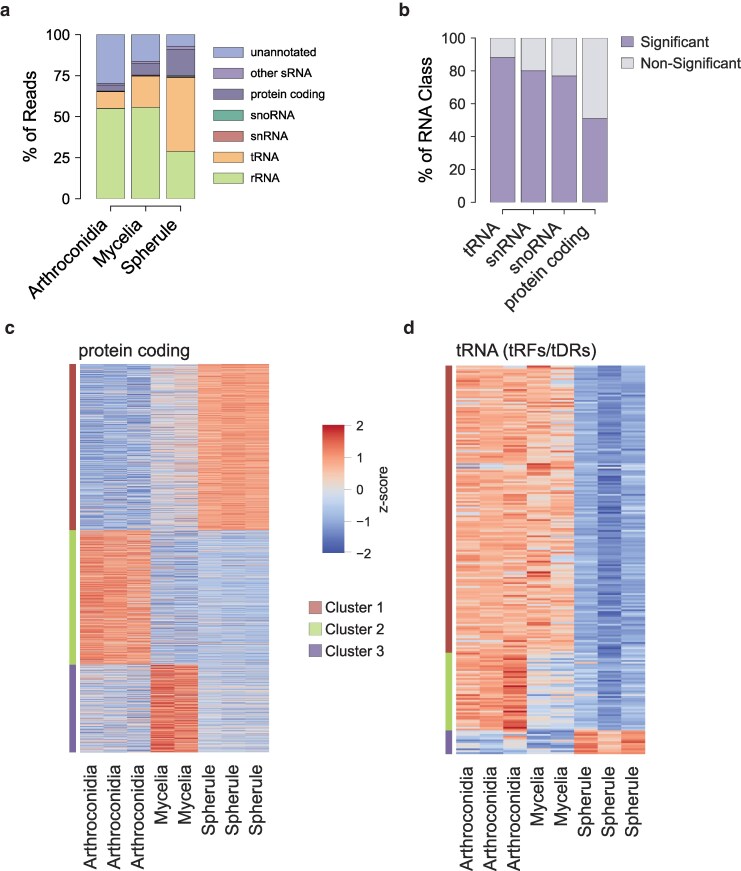
Differential expression analysis of annotated RNAs across *C. posadasii* morphologies. a) Bar plot showing the percentage of mapped RiboMarker small RNA reads categorized by RNA type annotations in *C. posadasii*. b) Bar plot showing the percentage of transcripts from different RNA classes found significantly enriched (padj < 0.01) in a *Coccidioides* morphology. c and d) Hierarchical clustered heatmaps of protein-coding RNA abundance (b; 3,474 significantly different [padj < 0.01]) and tRNA fragments (c; 215 significantly different across time points [padj < 0.01]) in RiboMarker intracellular small RNA sequencing datasets. The color gradient represents Z-scores, with clusters indicated by colored bars on the left.

We observed differences in detected proportions of sRNA transcript types across sample types in the early time points (Fig. 2a). For example, we see small, but noticeable increases in tRNA-derived and protein-coding-derived sRNA detection in transitions between arthroconidia to mycelia. This may be indicative of similar small RNA expression levels among saprobic stage samples, but also reflecting differences with respect to morphology, especially with increases in transcription and translation. We also observed these same expression changes magnified with respect to spherules, suggesting a similar increase in gene expression which may be representative of transcriptomic and translational changes during the transition to the parasitic life cycle (Carlin et al. 2021). A deeper look at ncRNAs detected in our study suggests that as compared to protein-coding derived RNAs, of which only 50% appear to change in expression across all life cycle morphologies (Fig. 2b; “protein coding”), we find that nearly 80% or more of annotated ncRNAs subtypes detected in our data show perturbations. This suggests large transcriptomic profile modulations, coding and noncoding, are a defining characteristic of morphologies in *Coccidioides*.

An assessment of the abundance of small RNA molecules derived from mRNAs across our samples identified 3,641 that significantly fluctuated during the time course (Supplementary File 2). A heatmap of these is presented in Fig. 2c (data in Supplementary File 2) where we observed limited variation in transcript abundance among replicates for each morphology. Notable patterns of expression were made evident through hierarchical clustering of these data where we were able to define 4 clusters specific to the arthroconidia (Fig. 2c, cluster 2), mycelia (Fig. 2c, cluster 3), and spherules (Fig. 2c, cluster 1) ecotypes. From each of these clusters, we isolated representative examples which highlight the differences in mRNA-derived small RNAs abundance across time points (Supplementary Fig. 6). For example, we saw a relatively high abundance of small RNAs generated from the gene INDA1 specific to mature mycelia (Supplementary Fig. 6, cluster 3). This gene encodes an amino acid permease, responsible for, among other processes, the uptake of amino acids from the environment (Wipf et al. 2002; Cappellazzo et al. 2008). Regarding spherule enriched expression, we saw an increased capture of reads derived from HSK1 (Supplementary Fig. 6, cluster 1), which encodes a serine-threonine kinase, whose homolog has been shown to regulate DNA replication initiation in fission yeast (Shimmoto et al. 2009), a process known to be highly upregulated in the spherule morphology (Ward and Angert 2008). Finally, we identified a transcript differentially expressed along the arthroconidia to mycelia transition that encodes 4-hydroxyphenylpyruvate dioxygenase (HPPD; Supplementary Fig. 6, cluster 2), a protein responsible for the metabolism of tyrosine and phenylalanine as a carbon source (Wang et al. 2022).

Another subtype of RNA that revealed substantial changes in abundance was fragments derived from transfer RNAs (or tRFs/tDRs). A heatmap depicting the expression levels of differentially expressed tRNA-derived RNAs is presented in Fig. 2d (data in Supplementary File 3). Along with the high degree of reproducibility among morphological replicates, we also noted that the abundance of tRNA fragments was generally high in the arthroconidia and mycelia relative to the spherules (Fig. 2d, clusters 1 and 2). This may be reflective of the observed differences in protein synthesis among morphologies (Viriyakosol et al. 2013). By and large, spherules showed low levels of tRNA fragment abundance (Fig. 2d, cluster 1 and 2, spherules samples) compared to arthroconidia and mycelia, suggesting a higher requirement for full-length tRNAs to regulate protein synthesis (Torrent et al. 2018). Interestingly, spherules contained a small cluster of tRNA fragments that were specifically abundant (Fig. 2d, cluster 3), including an abundance of glycine and tyrosine tRNA 3′ halves, histidine 5′ halves, and serine 3′-derived fragments (Supplementary Fig. 7). While previous literature has shown tRNA fragmentation to globally regulate protein translation, mRNA stability, and RNA binding protein activity (Liu et al. 2021), their functions in fungi have remained elusive.

### Transcriptomic analysis reveals distinct populations of unannotated small RNA producing loci

**Fig. 3.**
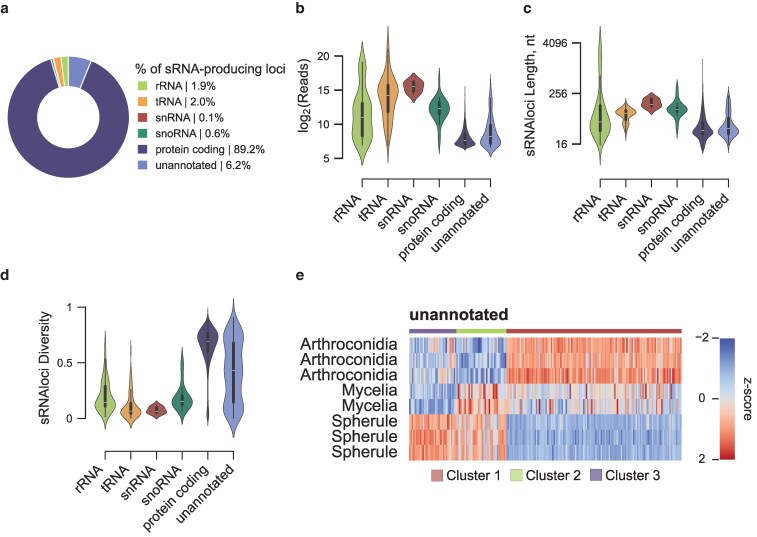
Characterization and differential expression of unannotated RNA loci across *C. posadasii* morphologies. a) Pie chart illustrating the distribution of ShortStack clusters within each annotated RNA type or if it fell within an unannotated region. b/c/d) Violin plots showing the b) log_2_ average read counts of mapped ShortStack loci by RNA type; c) length of ShortStack loci identified among the different RNA classes; d) diversity of reads within each ShortStack loci. e) Clustered heatmap of unannotated ShortStack loci showing significant differential expression across time points (padj. < 0.01) in both intracellular small RNA samples. Cluster groups are indicated by colored bars at the top.

Beyond the confines of annotated RNAs derived from the *Coccidioides* genome, our transcriptional data suggests a small but significant population of RNA is derived from unannotated (or intergenic) regions (Fig. 2a). To identify potential novel RNA producing loci, we used reference-aligned, RiboMarker RNA sequencing reads as input into ShortStack (see Materials and methods) (Axtell 2013), a bioinformatics tool that performs comprehensive de novo annotation and quantification of inferred small RNA genes (regardless of annotation) and fragment-generating loci. These ShortStack-inferred RNA loci were then mapped against the *C. posadasii* genome to assess where they land relative to its annotated and unannotated regions. Results from our ShortStack analysis revealed that a subset of small RNA read clusters were derived from rRNAs and tRNAs (Fig. 3a), while most inferred clusters were found in protein-coding genes (89.2%), with unannotated regions comprising the second highest population (6.2%; Fig. 3a). Granted, while clusters mapping to rRNA and tRNA genes are high in average read count (Fig. 3b), which is to be expected, unannotated make up a smaller but more diverse cohort of detected RNAs (Fig. 3d). While this RNA sub-population is derived from previously unannotated noncoding regions of the genome, this phenomenon is found across all branches of life (Argaman et al. 2001; Kapranov and St Laurent 2012), including fungi (Gervais and Shapiro 2024). Identification of these seemingly novel RNAs is further complicated by the current state of genomes from *Coccidioides* and other fungi, as they remain poorly characterized, leading to less properly annotated coding and noncoding genes. Therefore, to further characterize these unannotated-derived RNA species, we took a data-driven, unbiased approach to identify loci of unannotated RNA expression across the morphologies of *Coccidioides* central to this study.

To further characterize the unannotated loci from our RiboMarker *Coccidioides* small RNA libraries, we defined them using criteria described in Dorga et al. (Dogra et al. 2024) (Materials and methods and Fig. 3b to d) using the following metrics: locus read abundance (Fig. 3b), locus length (Fig. 3c), and locus read diversity (Fig. 3d). We observed significant differences (Fig. 3b and c; *P* < 0.0001; Materials and methods) in locus abundance and length when comparing annotated noncoding RNAs (ribosomal RNAs [rRNAs], transfer RNAs [tRNAs] small nuclear RNAs [snRNAs], and small nucleolar RNA [snoRNAs]) to both protein-coding-derived and unannotated RNA loci. This is reflective of both the bias of the library preparation for smaller RNAs and expression differences, especially between protein-coding and noncoding RNAs highlighted here. Interestingly unannotated clusters, along with protein-coding loci, show higher diversity (Fig. 3d; pval. 1 × 10-253), compared to annotated ncRNA clusters. These data suggest that ShortStack small RNA loci that fall within annotated regions (such as protein-coding genes) generally show less diverse coverage than unannotated loci, which may be representative of a lower-complexity region or high-specificity in fragments that are generated from ncRNAs in general. Additionally, unannotated genes may, in part, reflect both coding and noncoding genes that are yet to be annotated, possibly explaining why unannotated regions are “diverse” in read diversity.

Similarly for annotated transcripts, we identified RNAs produced from these novel loci that significantly fluctuated during the life cycle of *C. posadasii* and can be found in Fig. 3e (data in Supplementary File 4). As with annotated regions, hierarchical clustering revealed subsets of these transcripts that exhibit morphology-specific expression (Fig. 3e, clusters 1 to 3; logFC2 values for this heatmap can be found in Supplementary File 3). Focusing on the spherule morphology, we highlighted 2 examples (of many), which showed high levels of enrichment in this parasitic growth phase (Supplementary Fig. 8). For example, downstream of CPSG_01053 (predicted to encode a CORD and CS-domain containing protein) (Zhang et al. 2010), we identified sRNAlocus_4461 a novel small RNA-producing loci (Supplementary Fig. 8a) whose expression is derived from the opposite strand of the neighboring gene. RNACentral (The RNAcentral Consortium 2019) and nucleotide BLAST (Sayers et al. 2024) sequence analyses of this (and fragments of this) locus returned no significant similarities to any known annotated RNA, fungal or otherwise, but rather a collection of noncoding and hypothetical RNAs (data not shown). An RNAfold analysis (Lorenz et al. 2011), however, revealed a potential secondary structure with a minimum free energy of −26.20 kcal/mol (Supplementary Fig. 8b) representing a sequence from the most highly expressed regions of this cluster (Supplementary Fig. 8a; highlighted red box). For context, analysis of 20 randomly generated RNA sequences with identical %GC content yielded an average of −15.97 kcal/mol with a standard deviation of ±3.80 (data not shown). Another example can be found downstream of 2 protein-coding genes (on opposite strands), CPSG_03826 (encoding a 3-hydroxyisobutyryl-CoA hydrolase), an enzyme responsible for amino acid and fatty acid catabolism (Maggio-Hall et al. 2008), and CPSG_03827 (encoding a predicted DUF1264-domain containing protein), a protein domain found in enzymes involved in polycyclic aromatic hydrocarbon biodegradation and histone acetylation (Tello-Ruiz et al. 2022). Additionally, we identified sRNAlocus_4580, which potentially encodes 3 separate small RNA-producing loci of varying lengths (Supplementary Fig. 8c) expressed in both the 96-h mycelia time point and in spherules. Again, RNACentral (The RNAcentral Consortium 2019) and nucleotide BLAST (Sayers et al. 2024) sequence analyses of this (and apparent fragments of this) cluster returned no significant similarities to any known annotated RNAs. An RNAfold analysis (Lorenz et al. 2011) of the 2 small RNA producing sections of sRNAlocus_4580 (Supplementary Fig. 8c; highlighted red boxes) as well as the entirety of sRNAlocus_4580 revealed 3 intriguing potential secondary structures with a minimum free energy of −25.00, −23.10, and −53.40 kcal/mol, for Supplementary Fig. 8d to f, respectively.

While these data should not be taken as definitive proof of unannotated RNAs functionality, their putative structure and morphology-specific expression may suggest regulation of their expression and downstream utilization. To further validate the presence and expression patterns of unannotated RNAs across morphologies, we performed additional total RNA sequencing, (which includes RNAs from both small and long [>200 nt] species), to detect example RNAs presented herein (or their parental RNAs) as well as assess the correlation of unannotated small RNA expression to total RNA expression. In general, when comparing RiboMarker-enhanced libraries to total RNA sequencing libraries derived from the same sample, consistently high correlation was found (Supplementary Fig. 9) regardless of same-sample comparison. Additionally, we were able to validate that all example RNA loci shown herein were recapitulated in total RNA data, at least in the context of expression from said loci (data not shown). Taken together, these, and other, unannotated small RNA loci may contain functional elements, either consisting of yet-to-be canonical noncoding RNAs or potential microRNA-like RNAs (Lee et al. 2010; Cui et al. 2019). Further work will be required to validate and ascertain the functions of these putative small RNAs.

### Differential fragmentation patterns of annotated and unannotated noncoding RNAs correlate with *Coccidioides* morphology

**Fig. 4.**
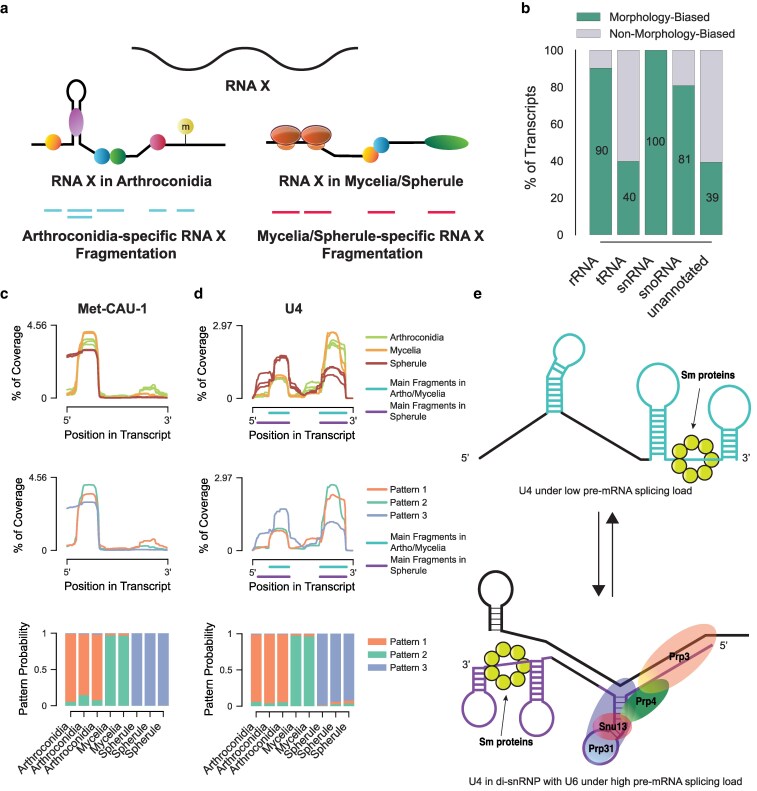
Analysis of RNA fragmentation patterns in different *Coccidioides* morphologies. a) Schematic representation of an RNA exhibiting differential fragmentation patterns in arthroconidia vs mycelia/spherules forms. b) Bar chart depicting the percentage of transcripts from different RNA classes, with and without significant morphology bias (padj < 0.05). c to e) Graphs showing the percentage of coverage across the transcript positions for 3 different RNA samples: Met-CAU-1 c), U4 d), and sRNALocus_4698 e) in arthroconidia (orange), mycelia (green), and spherules (blue) forms, along with 3 modeled patterns of fragmentation (middle). Below each coverage graph, bar charts represent the calculated probability of observing each of the 3 fragmentation patterns across different morphological states.

Previous literature has utilized RNA expression/abundance (Sonawane et al. 2017) and modification patterns (Gilbert and Nachtergaele 2023; Ando et al. 2024) as a means of categorizing biological sources, such as tissue identification or diseased vs healthy samples (Jonkhout et al. 2017), among others. While the molecular function of RNA fragments is poorly understood, data suggests these may, in part, be representative of protein-bound protected regions of RNA species, evidence of secondary structure, or regulated effectors of gene expression (Ule et al. 2005; Underwood et al. 2010; Li et al. 2022). Together, these data suggest that perturbations to cellular homeostasis or development-related gene expression, may, in turn, result in unique RNA fragment profiles reflective of these changes. For example, as shown in Fig. 4a, assuming the same transcript in 2 different fungal morphologies, interactions with RNA binding proteins, modifications states of the RNA, flexible secondary structures, or regulated translation of mRNAs, one may hypothesize that these cis- and trans-acting effectors could lead to different processing or degradation of a given RNA. Therefore, we questioned whether RNA fragmentation patterns could be used as a classification system to identify *Coccidioides* morphologies.

Given the myriad regulatory elements that regulate life cycle transitions between saprobic to parasitic (Whiston et al. 2012), and as a result, may alter the fragmentation of *Coccidioides* RNAs, we compared RiboMarker-enhanced small RNA sequencing libraries of arthroconidia, mycelia, and spherule morphologies (Fig. 2a). Briefly, ncRNAs identified using tRNAScanSE and RFAM, or unannotated sRNA-producing loci identified using ShortStack, were used for differential fragmentation profile analysis. Of these molecules we found that, more often than not, a transcript exhibited a fragmentation pattern significantly biased to different morphologies using our set of annotated and unannotated loci (Fig. 4b).

Two examples of these morphology-biased fragmentation profiles are shown in Fig. 4c and d. Figure 4c shows the RNA fragmentation profile from arthroconidia, mycelia, and spherules across tRNA-methionine (Met)-CAU-1, a transfer RNA responsible for decoding of Methionine codons found in mRNA during protein translation and often involved in translation initiation (Cigan et al. 1988). Transcript coverage (top panel) reveals differential fragmentation patterns near the 3′ end of the tRNA in both arthroconidia and mycelia morphologies, while absence of these fragments in spherule. Additionally, 5′ fragment coverage in the spherule is more robust starting at the 5′ terminal base compared to arthroconidia and mycelia, which show coverage beginning a few bases downstream. Using a machine learning approach, we categorized these read pileups into 3 distinct patterns to capture these data (middle panel). Notably it appeared that these patterns were representative of the 3 distinct *Coccidioides* morphologies (top and middle panels). We sought to probabilistically determine if there was any association between the 3 morphologies and the 3 RNA fragmentation patterns by fitting each individual replicate to the representative model generated for these transcripts (top fit to middle). Pattern membership probabilities were generated for each sample (bottom panel) where we observed perfect correlation between *Coccidioides* morphology and the associated RNA fragmentation patterns for tRNA-Met-CAU-1. Similarly, we observed this phenomenon regarding the U4 snRNA is shown in Fig. 4d. Here, we saw an increased complexity of fragmentation compared to tRNA-Met-CAU-1, where the ratio of 5′ and 3′ derived fragments is modulated relative to morphology type. In spherules, we observe a more even distribution of fragments derived from the 5′ and 3′ end of U4, while arthroconidia and mycelia show more 3′ bias to fragment generation (Fig. 4d; top panel). Again, these differential fragmentation profiles allow us to delineate between replicates based on associated morphologies, suggesting these profiles uniquely represent their associated morphology (Fig. 4d; bottom panel). Furthermore, this pattern of fragmentation of U4 snRNA is supported by data comparing the relative pre-mRNA processing load differences across *Coccidioides* morphologies. Previous research has observed relatively lower protein-coding expression in arthroconidia and mycelia ecotypes compared to spherule (Carlin et al. 2021), where transcription is significantly increased to support its parasitic morphology (Whiston et al. 2012). We hypothesize that, in the specific case of U4 snRNA, these fragment patterns may be, in part, reflective of increased intra- and intermolecular interactions of splicing machinery with respect to modulation of splicing requirements. Thus, in the case of low splicing load (e.g. relatively low levels of transcription) it would be expected that U4 snRNA is less likely to be in complex with U6 snRNA (to form a di-snRNP) as well as associated splicing factors (Fig. 4e; top illustration). In instances of high splicing load (e.g. relatively high levels of transcription) U4 is more likely forming di-snRNP with U6 snRNA and other splicing factors in formation of the B complex of the spliceosome (Fig. 4e; bottom illustration) (Munding et al. 2013; Hardin et al. 2015). But while the change in fragmentation patterns between spherule and arthroconidia/mycelia in the context of U4 snRNA do speak to this hypothesis, additional experiments would be required to show a causal effect between changes in RNA:RNA and RNA:protein interaction and the resulting fragmentation patterns we see in this sequencing data. Given the unique biology of *Coccidioides* and the limited availability of functional genetic studies, we emphasize that these clustering analyses identify correlative patterns rather than definitive regulatory pathways. Although we cannot conclude that fragmentation of these highlighted RNAs drives morphological changes, these machine learning–derived associations provide a valuable framework for hypothesis generation. Pending experimental validation, morphology-specific sRNAs and RNA fragment profiles should be interpreted as candidate biomarkers, particularly those enriched in the parasitic spherule form.

### Cell-free and exosomal small RNA pools contain putative effectors of cell–cell and pathogen-host communication

Upon infection, fungal pathogens can modulate the host immune response to generate a preferred microenvironment to promote pathogenesis (Lai et al. 2023b). This is often achieved through the export of molecular effectors (e.g. proteins, RNAs, etc.) packaged in exosomes, a sub-population of EVs with the ability to interact and fuse with lipid bilayer (Liebana-Jordan et al. 2021), facilitating cross-kingdom communication with host cells (Cheng et al. 2023). Exported RNA effectors are known to, in part, target RNAi machinery in host cells to selectively silence target genes (Bitencourt et al. 2022), which can lead to a dampened or neutralized host response, allowing the fungal pathogen to persist and propagate within the host (Liu and Hu 2023). While these processes are well known in plant-associated fungal pathogens (Zhou et al. 2022), little is known about the RNA content of the extracellular environment of *Coccidioides*. Therefore, we sought to leverage sequencing data from our extracellular fractions to determine what, if any, RNA exists, or may be actively exported, outside of the cell, and how this may change across *Coccidioides* morphologies.

To explore the potential of extracellular RNA related to *C. posadasii*, we performed RiboMarker sequencing on exosome- and cell-free-associated RNAs isolated from fractions of filtered, conditioned media used in the growth and propagation of the arthroconidia, mycelia, and spherules samples. To control for background RNA not derived from the fungus, we prepared media-only (Control) samples for both exosomal and cell-free RNA fractions at all morphological stages (see Materials and methods). Because only a single biological replicate was sequenced for the extracellular fractions, differential abundance analyses should be considered exploratory. Moreover, despite the inclusion of media-only controls, distinguishing active, targeted EV export from passive RNA release (e.g. due to cell lysis, mechanical disruption, or culture stress) remains technically challenging. This is further complicated by difficulties in standardizing living biomass across morphologically distinct fungal forms, such as arthroconidia and spherules. Accordingly, these extracellular signatures represent a preliminary profile requiring further validation. Notably, initial analyses of sRNA gene classes revealed substantial differences in extracellular RNA composition among arthroconidia, mycelial, and spherule-derived cell-free fractions (Supplementary Fig. 10). To investigate the high degree of similarity in the RNAs detected in either the cell-free or exosomal fractions (Supplementary Fig. 10), we performed a transcript level analysis (Supplementary Fig. 11) where we found the 2 fractions were highly correlated with one another (*r* > 0.8), suggesting there are minimal discernable characteristics between the cell-free and exosomal RNA pools.

**Fig. 5.**
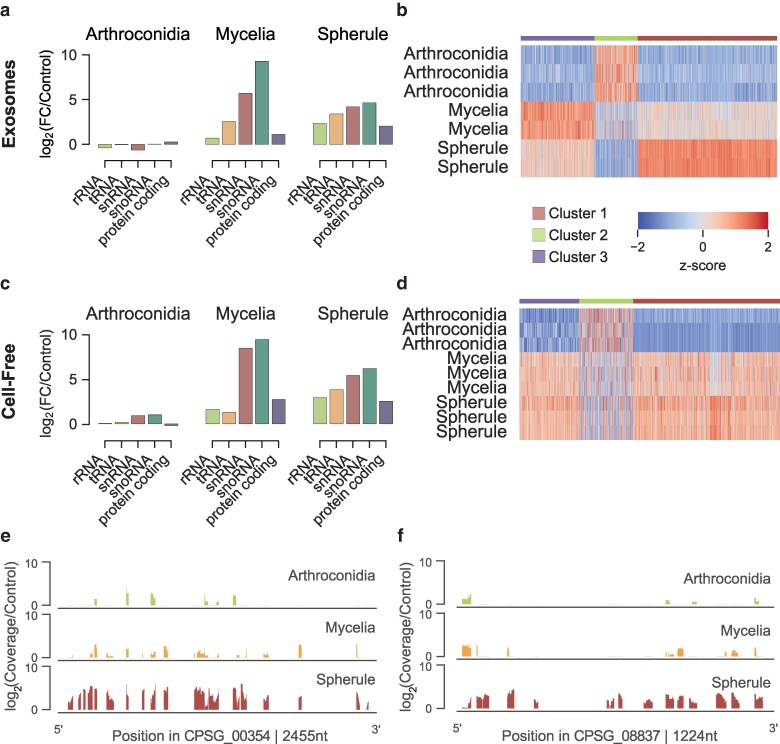
Extracellular RNA profiles vary across *C. posadasii* morphologies. a and c) Bar plots showing the enrichment over control of small RNA reads mapping to annotated RNA-generating loci for exosomal a) and cell-free c) fractions of conditioned media across *Coccidioides* morphologies. Clustered heatmaps of small RNA sequencing data from RiboMarker libraries derived from exosome- b) and cell-free- d) associated fractions. The exosome heatmap represents 1,239 RNAs, and the cell-free heatmap represents 2,651 RNAs, showing significant abundance differences across time points and spherule samples (padj < 0.01). e and f) Read coverage plots for 2 predicted genes, CPSG_00354 (e; predicted Heat Shock protein 70) and CPSG_08837 (f; predicted Enolase), illustrating the mapped read coverage of extracellular RNAs from exosomal samples at different stages.

Relative to our media controls, the distribution of RNA types across our different time points varied significantly (Fig. 5a and c). Among mycelia and spherules, we saw a >80-fold increase in snRNA-derived RNAs in the cell-free fraction and exosome fractions (Fig. 5a and c), with U2 snRNA-derived RNAs seeing the highest mean enrichment relative to their respective controls. This marked extracellular increase may be indicative of enhanced intracellular splicing activity and could be associated with global transcriptomic reprogramming as the fungus prepares for further growth and/or differentiation (Muzafar et al. 2021). In the subsequent transition from the mycelia to spherules, we noted a substantial shift: the abundance of snRNA-derived reads dropped by half, while there was a notable increase in tRNA-derived reads (Fig. 5c), which was also mirrored in the exosome fractions (Fig. 5a). This increase in tRNA fragments might be indicative of stress response and adaptation mechanisms associated with environmental changes during formation of spherules or could reflect increased protein synthesis (Nawrot et al. 2011; Li et al. 2022).

Clustering of expression data from both cell-free and the exosome fractions suggested distinct RNA populations were significantly (padj < 0.01) enriched relative to the *Coccidioides* morphology (Fig. 5b and d). For the exosome samples, we saw a small population of RNAs enriched relative to the mycelia and spherules (Fig. 5b; cluster 1). Interestingly, a significant majority of extracellular small RNA and RNA fragments were found present in higher abundance in both the mycelia and spherule exosomal fractions (compared to arthroconidia), with cluster 1 enriched in spherules and cluster 3 more abundant in the mycelia (Fig. 5b; clusters 1 and 3; Supplementary File 5). The cell-free fraction highlighted similar patterns of RNA enrichment (Fig. 5d; Supplementary File 6), with clusters 2 and 3 containing highly abundant molecules in both mycelia and spherules, whereas cluster 1 seems more highly enriched in arthroconidia, however, not as distinctly as the exosome fraction. Together, these data suggest that the extracellular RNA content suggest that the life cycle and metabolic states of *Coccidioides* may play a role in regulating RNA export *en masse*.

To gain more perspective on the biological function of the pool of extracellular RNAs, we focused on the exosome fraction, specifically those RNAs found specifically enriched in spherules (cluster 1) (Fig. 5b). In these data, we observed 2 intriguing examples of enriched small RNAs derived from protein-coding genes that have been previously shown in the literature to associate with EVs (Liebana-Jordan et al. 2021). For example, small RNAs derived from CPSG_00354, encoding a predicted Hsp70-like protein, show an 8-fold increase in abundance in the spherule exosome fraction relative to the control and time course samples (Fig. 5e). Interestingly, Hsp70 domain-containing protein motifs, found in protein chaperones and known virulence factors in pathogenic fungi (Silveira et al. 2013; Horianopoulos and Kronstad 2021), have been shown to be the only universal factor found across EVs tested from a panel of 6 different fungal pathogens (Parreira et al. 2021), suggesting that heat-shock related proteins could play a universal role in fungal EV biogenesis and host interactions (Silveira et al. 2013). Another example was the presence of small RNAs derived from a predicted enolase gene (Fig. 5f) enriched in the spherule morphology. The glycolytic enzyme product of this protein-coding gene has been previously shown to be a major constituent of fungal EVs (Nimrichter et al. 2016) and has previously been used as a successful antigen in vaccination models in mice for *C. albicans* infections. It is important to note that we are only detecting associated mRNA fragments to these proteins, and proteins themselves can be loaded into EVs without mRNAs present. Whether protein translation occurs in EVs is currently unknown; however, there is evidence that human exosomes transport mRNA fragments that may act as competing RNA to regulate gene expression in recipient cells (Batagov and Kurochkin 2013). While more evidence would be required to confirm functions of mRNA-derived RNAs in *Coccidioides* EVs, it offers a compelling hypothesis for these highlighted mRNA fragments and others we detected in this study.

Outside of mRNA-derived small RNAs, we also found specific tRNA fragments enriched in our extracellular spherule samples, including Leucine tRNA and Proline tRNA fragments (Supplementary Fig. 12). While their function outside of the cell is yet to be described, tRNA fragments like these have been shown to populate fungal EVs (Bitencourt et al. 2022), possibly playing roles in the regulation of host gene expression (Peres da Silva et al. 2015). More evidence is needed to determine the biological roles of these extracellular RNAs, outside of potential RNA biomarkers. Future directions could involve validating whether these extracellular RNAs, especially those enriched at certain life stages, play active roles in pathogenesis, signaling, or environmental adaptation in *Coccidioides*.

## Discussion

In this study, we present a comprehensive characterization of the small RNA landscape across key saprobic and parasitic morphologies of *Coccidioides*, providing new insight into RNA-mediated regulation during fungal development and pathogenesis. By applying RiboMarker-enhanced sequencing, we substantially expanded the repertoire of detectable small RNAs, uncovering RNA species derived from tRNAs, rRNAs, protein-coding genes, and previously unannotated genomic loci that are largely invisible to conventional small RNA sequencing approaches. Together, these data reveal extensive remodeling of small RNA populations across the *Coccidioides* life cycle and establish both intracellular and extracellular RNA signatures that correlate with distinct morphological states.

One of the most notable findings from this work is the discovery of hundreds of unannotated small RNA-producing loci, many of which exhibit expression levels and morphology-specific regulation comparable to annotated RNAs. While it is increasingly recognized that pervasive transcription from unannotated genomic regions is a common feature across eukaryotic genomes, including fungi, the functional significance of these RNAs remains poorly understood. The absence of predicted miRNA-like genes using tools such as miRDeep2 highlights a key limitation in current fungal RNA annotation pipelines and underscores the need for improved bioinformatic approaches tailored to fungal small RNA biology. It is likely that a subset of these unannotated loci represents previously undescribed noncoding RNAs, including snoRNAs, snRNAs, or fungal-specific microRNA-like RNAs, whose identification will require both methodological and conceptual advances in the field.

Integrating these newly identified sRNA populations with established biological pathways represents an important next step. The saprobic-to-parasitic transition in *Coccidioides* is strongly regulated by the transcription factor Ryp1, which coordinates extensive morphological and virulence-associated transcriptional programs (Mandel et al. 2022). It is therefore plausible that a subset of the differentially expressed sRNAs or unannotated RNA loci identified here intersect with Ryp1-regulated networks, either as downstream targets or as post-transcriptional modulators. However, in the absence of targeted functional validation—such as sRNA profiling in a *ryp1* deletion background or direct RNA-protein interaction assays—these relationships remain speculative. The clustering and expression patterns described here provide a framework for prioritizing such future mechanistic studies.

Genomic analyses indicate that *Coccidioides* species, like most fungi, encode the core components of an RNA interference (RNAi) pathway, including Argonaute and Dicer-like proteins (Torres-Martinez and Ruiz-Vazquez 2017). However, our data suggest that the extensive transcriptomic remodeling observed across morphologies is not primarily driven by canonical small interfering RNAs (siRNAs). In fungi, siRNAs typically mediate gene silencing and exhibit a strong 5′-uracil (U) bias reflecting Argonaute binding preferences (Lee et al. 2010). In contrast, the first-nucleotide composition of sRNAs in our dataset was relatively balanced (G: ∼35.9%, A: ∼26.5%, U: ∼20.5%, C: ∼17.1%), lacking a dominant 5′-U signature. The enrichment for 5′-G is consistent with a substantial contribution from ribosomal RNA fragments. Together, these observations suggest that, although *Coccidioides* retains RNAi capacity, the majority of morphology-associated sRNAs and RNA diversity captured here likely arise from alternative RNA processing, cleavage, or export pathways rather than canonical RNAi mechanisms.

Beyond cataloging RNA species, we introduce RNA fragmentation profiling as a complementary dimension for interpreting small RNA sequencing data. Our analysis demonstrates that both annotated and unannotated RNAs exhibit reproducible, morphology-specific fragmentation patterns. While these patterns do not imply causality in driving morphological transitions, their strong association with specific life stages suggests that RNA fragmentation reflects underlying cellular states, including transcriptional load, RNA-protein interactions, and post-transcriptional regulation. This proof-of-concept raises the possibility that RNA fragmentation signatures could be exploited as diagnostic or classificatory biomarkers, enabling identification of fungal states or infections without prior knowledge of pathogen identity.

A major contribution of this work is the characterization of extracellular RNA populations across *Coccidioides* morphologies. We demonstrate that both cell-free and EV-associated RNA pools exhibit distinct, morphology-dependent RNA signatures, including enrichment of snRNA- and tRNA-derived fragments. These findings are consistent with growing evidence that fungal pathogens export diverse RNA species into the extracellular environment, potentially mediating intercellular communication and host–pathogen interactions. Although some extracellular RNAs may arise from passive release during cell turnover, their reproducibility and morphology-specific enrichment argue for selective stabilization or export. Moreover, the detection of extracellular RNA fragments derived from genes previously linked to fungal virulence and EVs further supports the hypothesis that these RNAs may have functional roles or, at minimum, serve as robust biomarkers of infection.

While this study provides a comprehensive landscape of sRNA diversity across *Coccidioides* morphologies, several limitations should be considered. Notably, we utilized a *C. posadasii* chitinase knockout strain (Δ*cts2*/Δ*ard1*/Δ*cts3*), which enabled execution of the extensive RNA extraction and RiboMarker library preparation workflows outside of Biosafety Level 3 (BSL-3) containment. However, because chitinases play central roles in fungal cell wall remodeling, virulence, and developmental transitions (Hartl et al. 2011), their deletion could influence RNA-related processes. Indeed, *CTS2* and *CTS3* are required for completion of the parasitic lifecycle and proper endosporulation (Mead et al. 2020), raising the possibility that altered cell wall dynamics could indirectly affect intracellular RNA processing, stress-responsive transcriptional programs, or the efficiency and mechanisms of EV-mediated RNA export. Accordingly, while the morphology-dependent transcriptomic signatures and ∼500 unannotated loci identified here represent a robust and internally consistent resource, the precise patterns of RNA fragmentation and export may be partially shaped by the mutant background. Validation in wild-type strains will be necessary to determine the extent to which these profiles generalize to natural infection contexts. In addition, the exploratory nature of our extracellular RNA analyses—based on single biological replicates per condition—warrants caution. Future studies incorporating increased replication and rigorous biomass normalization will be required to definitively distinguish active vesicle-mediated RNA export from passive release mechanisms.

From a technical perspective, our study highlights the importance of library preparation chemistry in shaping biological conclusions drawn from small RNA sequencing data. The RiboMarker platform enabled inclusion of RNA species with diverse end chemistries and internal modifications, revealing layers of the transcriptome that are otherwise inaccessible. As interest grows in RNA-based diagnostics and biomarkers—particularly for infectious diseases where rapid, noninvasive detection is critical—the ability to comprehensively capture and interpret heterogeneous RNA populations will become increasingly important. In such contexts, biological origin or function of an RNA fragment may be secondary to its reliability as a detectable marker of disease state.

In conclusion, our findings provide a foundational atlas of intracellular and extracellular small RNAs across major *Coccidioides* life cycle stages and demonstrate that RNA expression and fragmentation patterns are tightly linked to fungal morphology. Beyond advancing our understanding of *Coccidioides* biology, these findings establish a framework for exploring RNA-based biomarkers of Valley fever and underscore the broader utility of enhanced small RNA sequencing approaches for studying fungal pathogens. As the incidence and geographic range of coccidioidomycosis continue to expand, resources such as this will be essential for guiding future diagnostic, mechanistic, and translational studies.

## Supplementary Material

jkag111_Supplementary_Data

## Data Availability

Datasets associated with this study are publicly available: raw RNA sequencing data files as well as small RNA count matrices (generated via counting reads overlapping annotated, or novel, features) are available at NCBI GEO: GSE290689. Supplemental material available at G3 online.
