# Supplementary material for: Transcriptomic characterization of Coccidioides morphological states using RiboMarker-enhanced RNA sequencing
Source: G3 (Bethesda). 2026 Apr 28;16(7):jkag111. doi: 10.1093/g3journal/jkag111 (PMC13334182; doi:10.1093/g3journal/jkag111)
Supplement: jkag111_Supplementary_Data [file jkag111_supplementary_data.zip › Supplemental_Figures_G3-2026-406762.pdf]

**Howard et al 2026**

**Supplemental Figures**

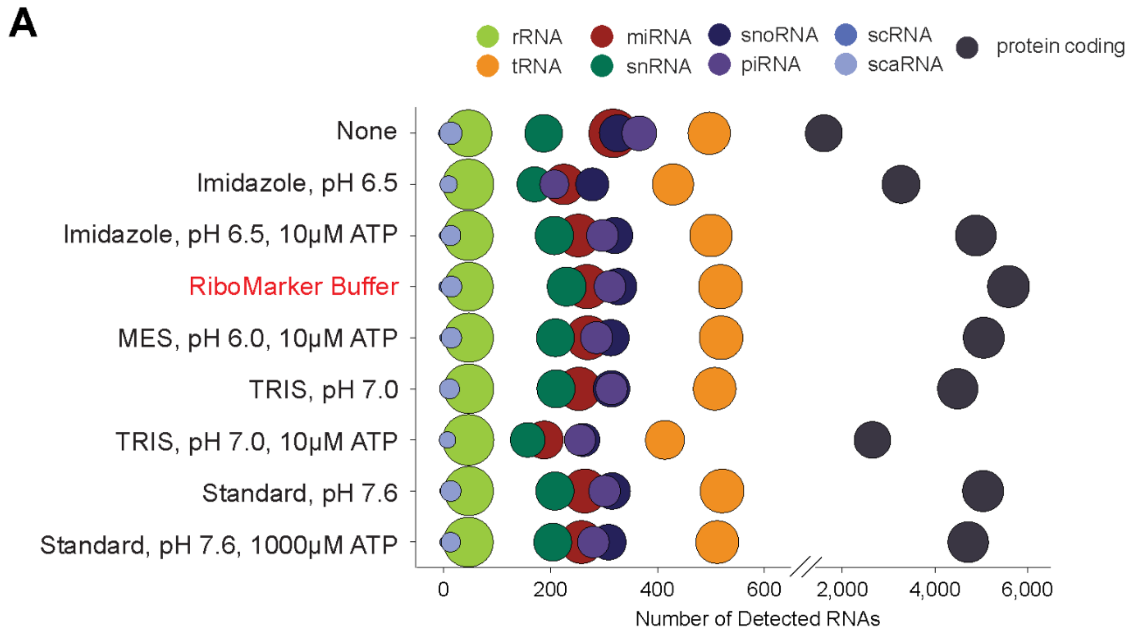


**Supplemental Figure 1**

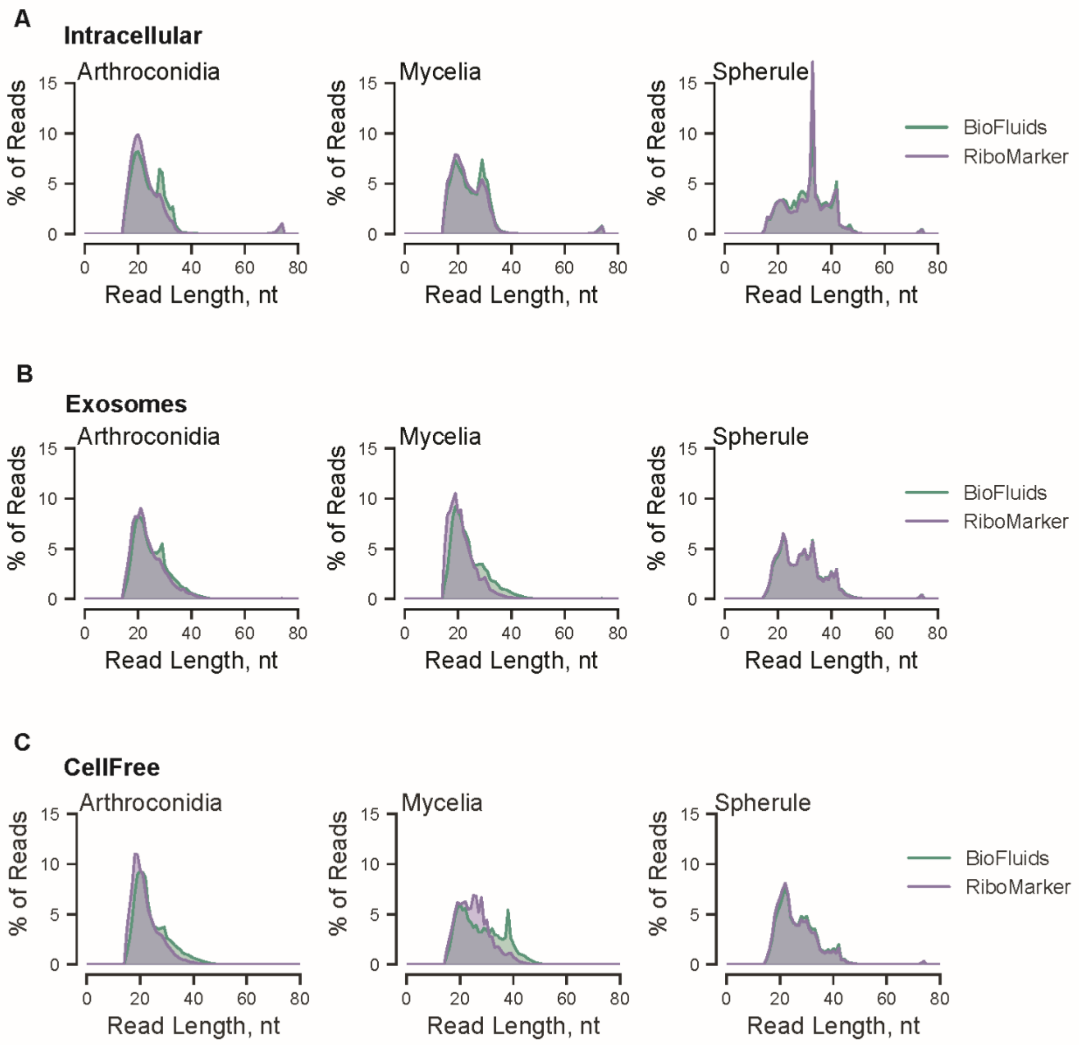


# Supplemental Figure 2

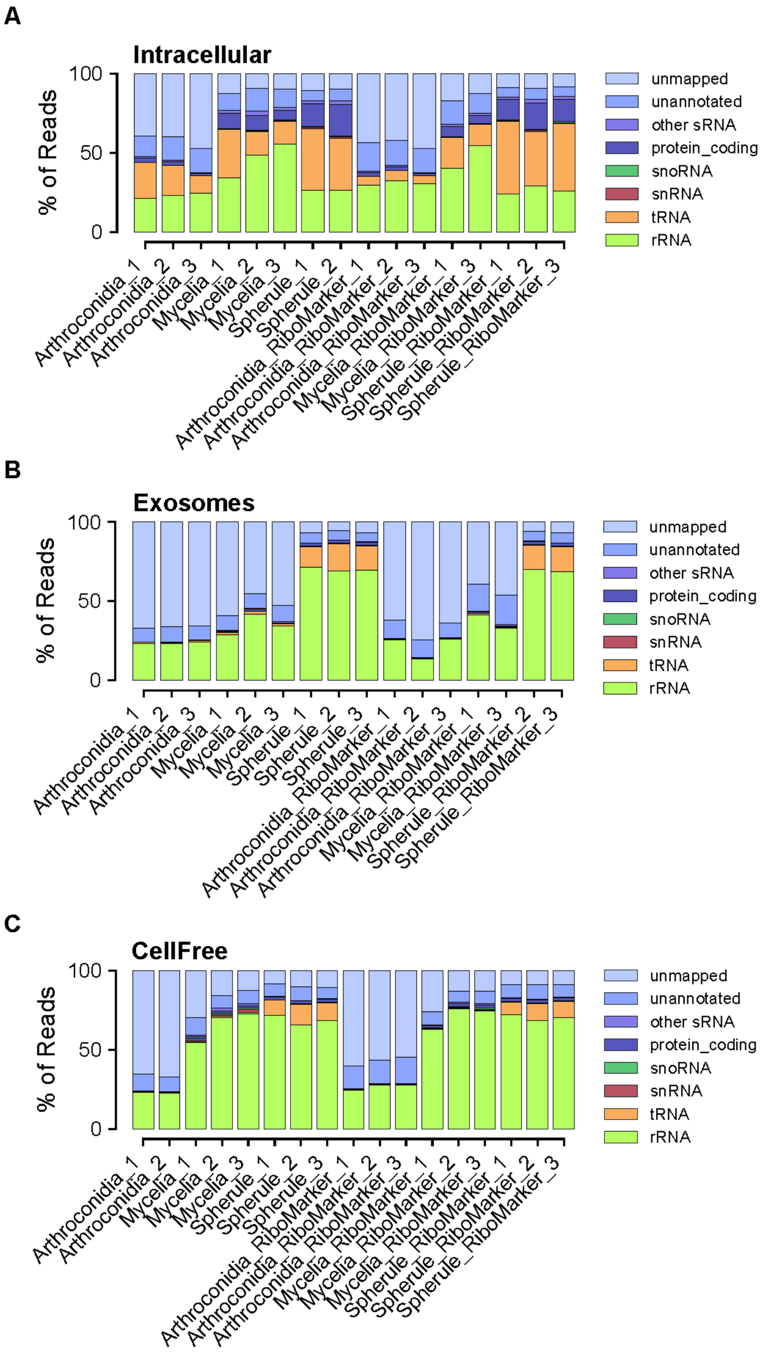


**Supplmental Figure 3**

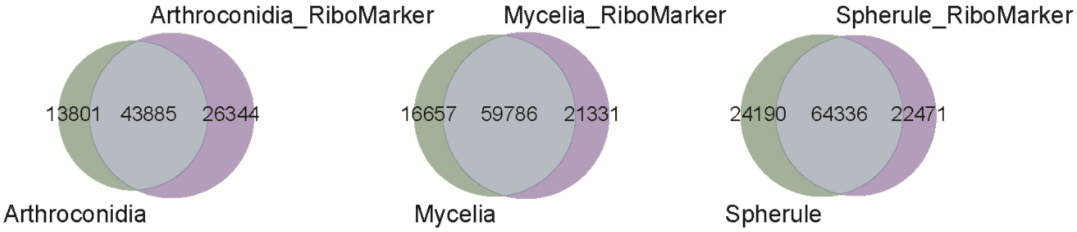


**Supplemental Figure 4**

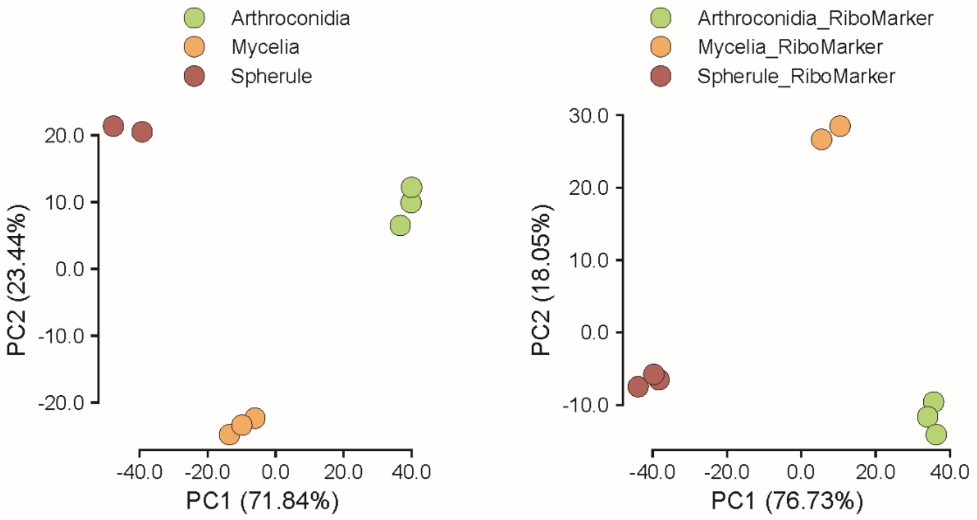


**Supplemental Figure 5**

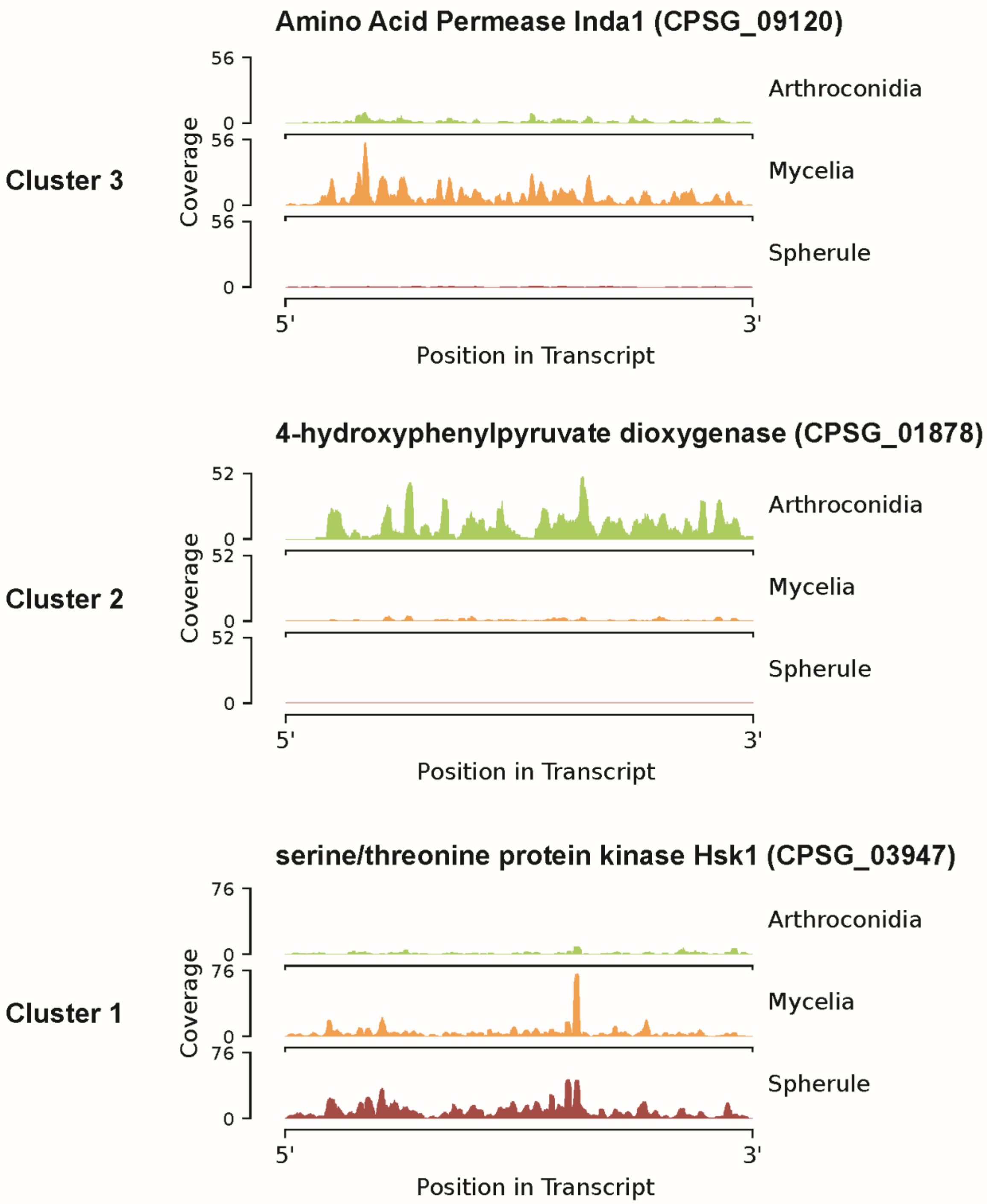


**Supplemental Figure 6**

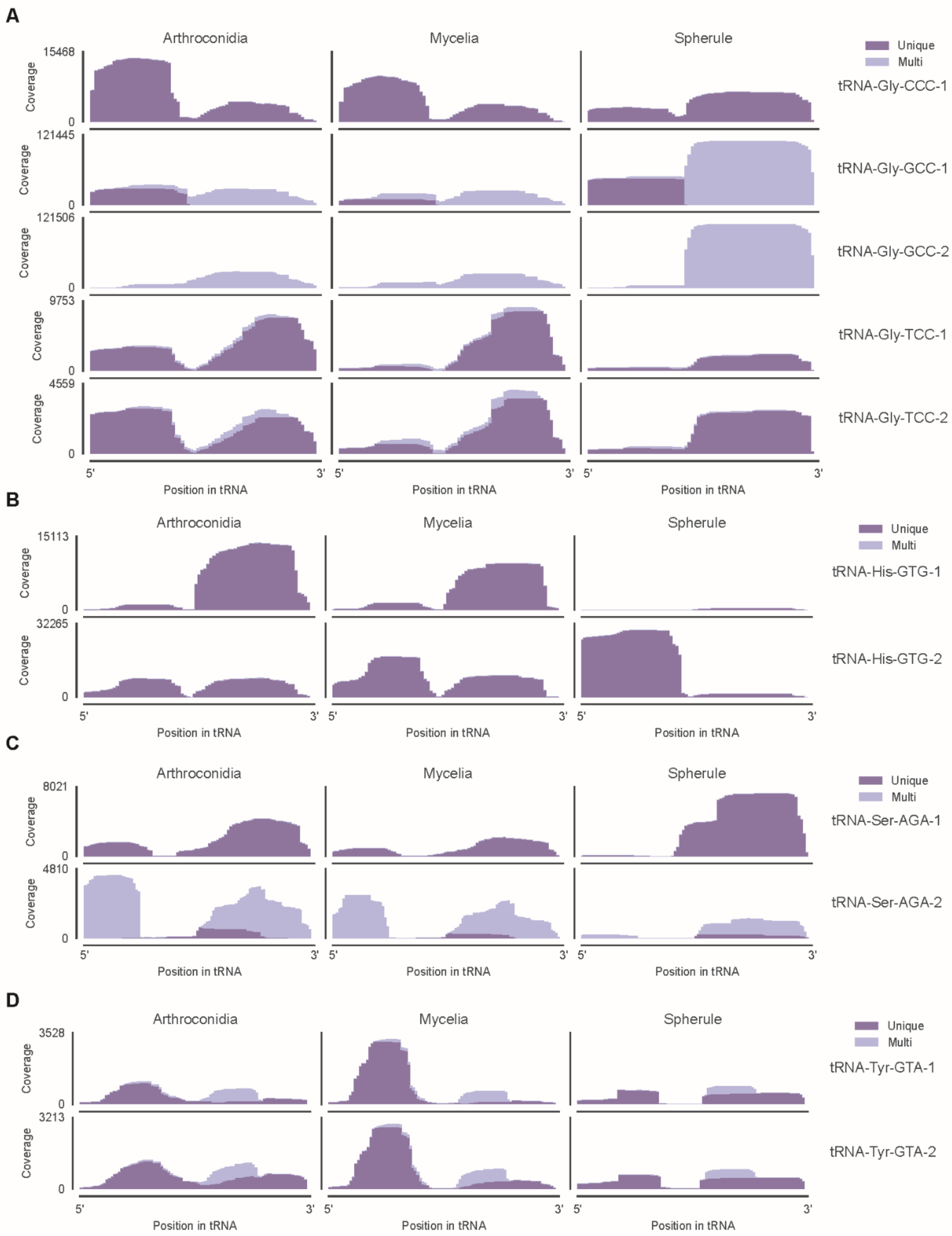


**Supplemental Figure 7**

**sRNAlocus_4461: Downstream and antisense to CORD and CS domain-containing protein (CPSG_01053)**

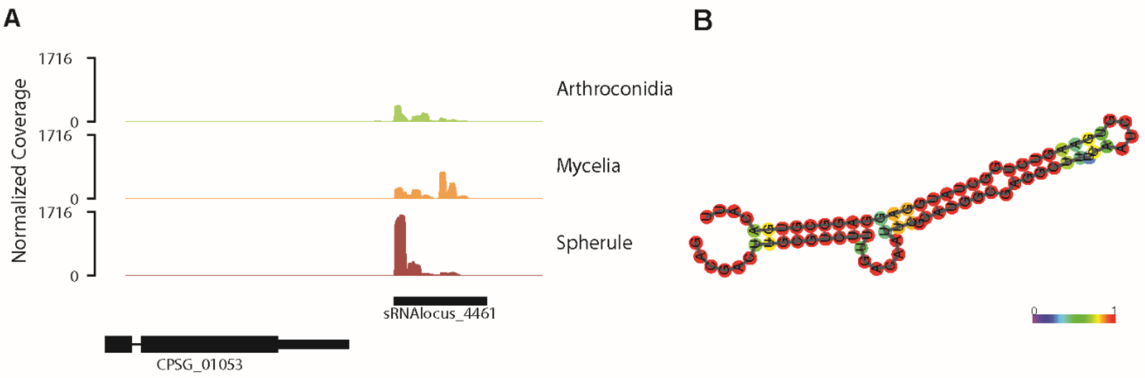


**sRNAlocus_4580: neighboring a DUF1264-domain containing protein (predicted)**

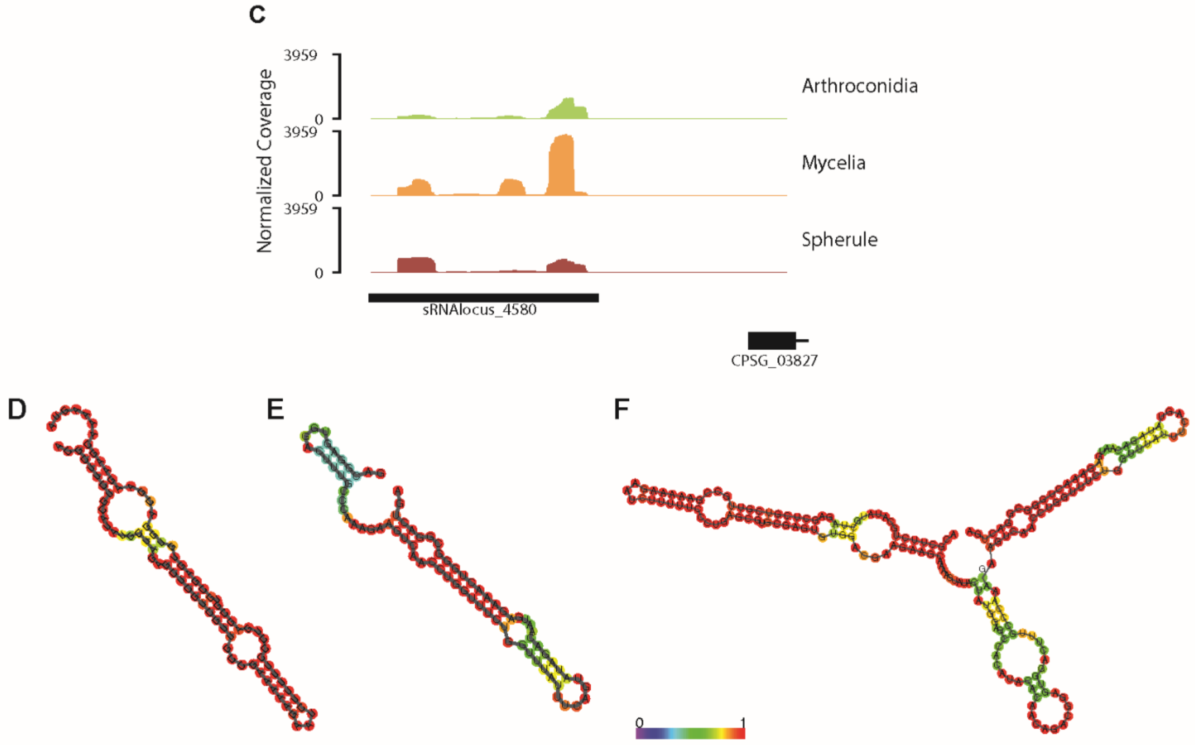


**Supplemental Figure 8**

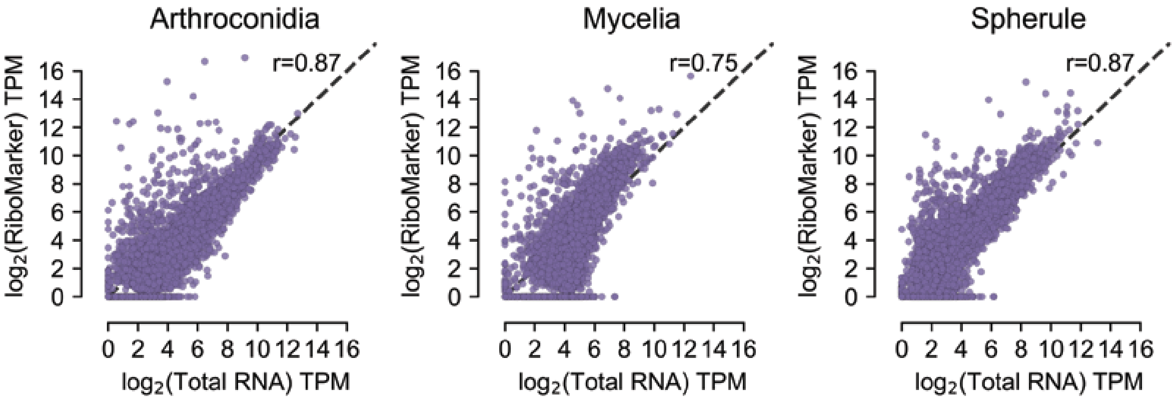


**Supplemental Figure 9**

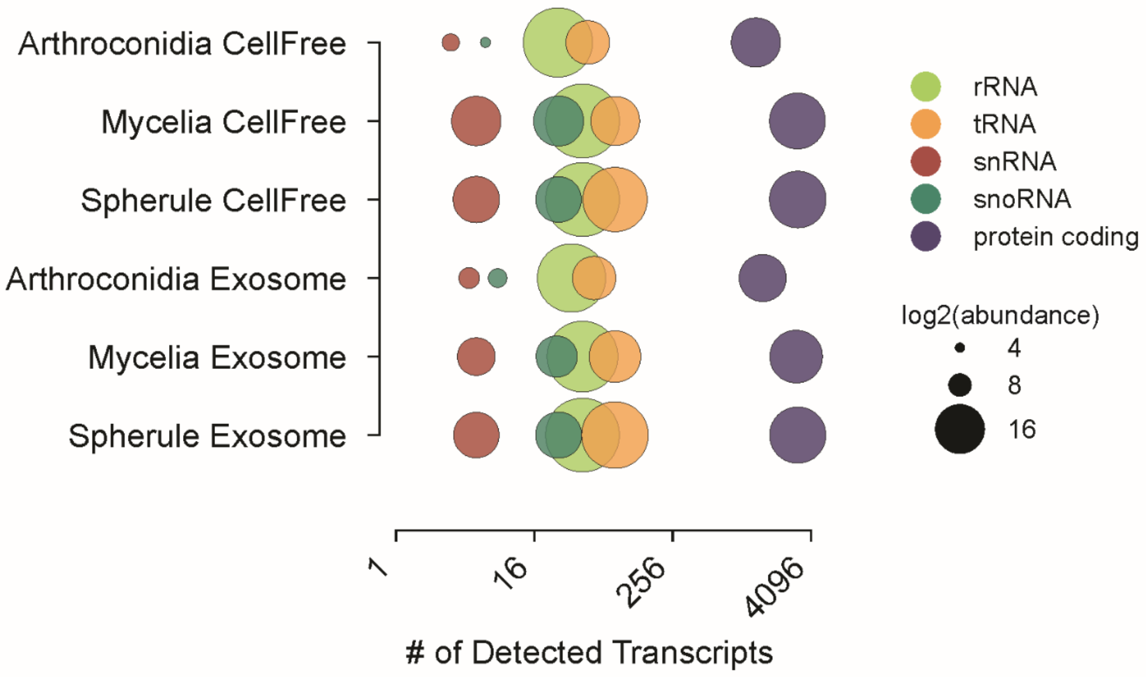


**Supplemental Figure 10**

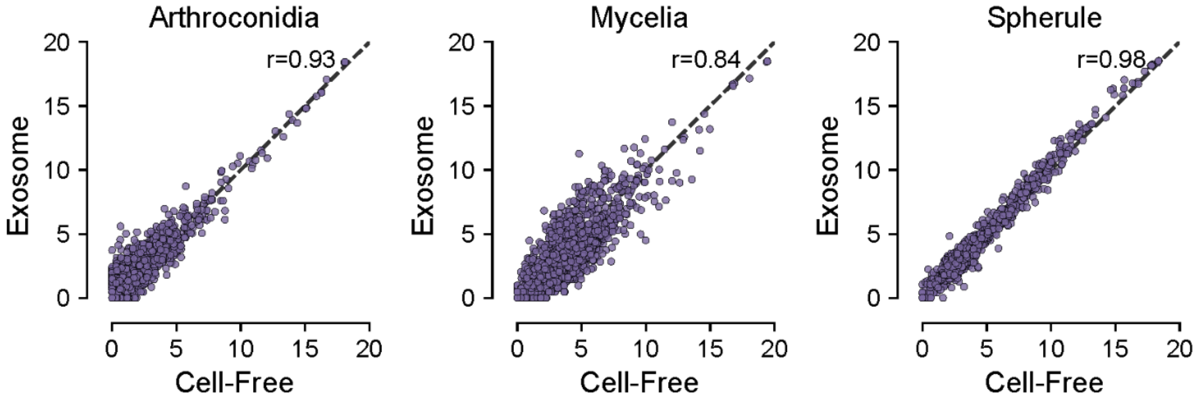


**Supplemental Figure 11**

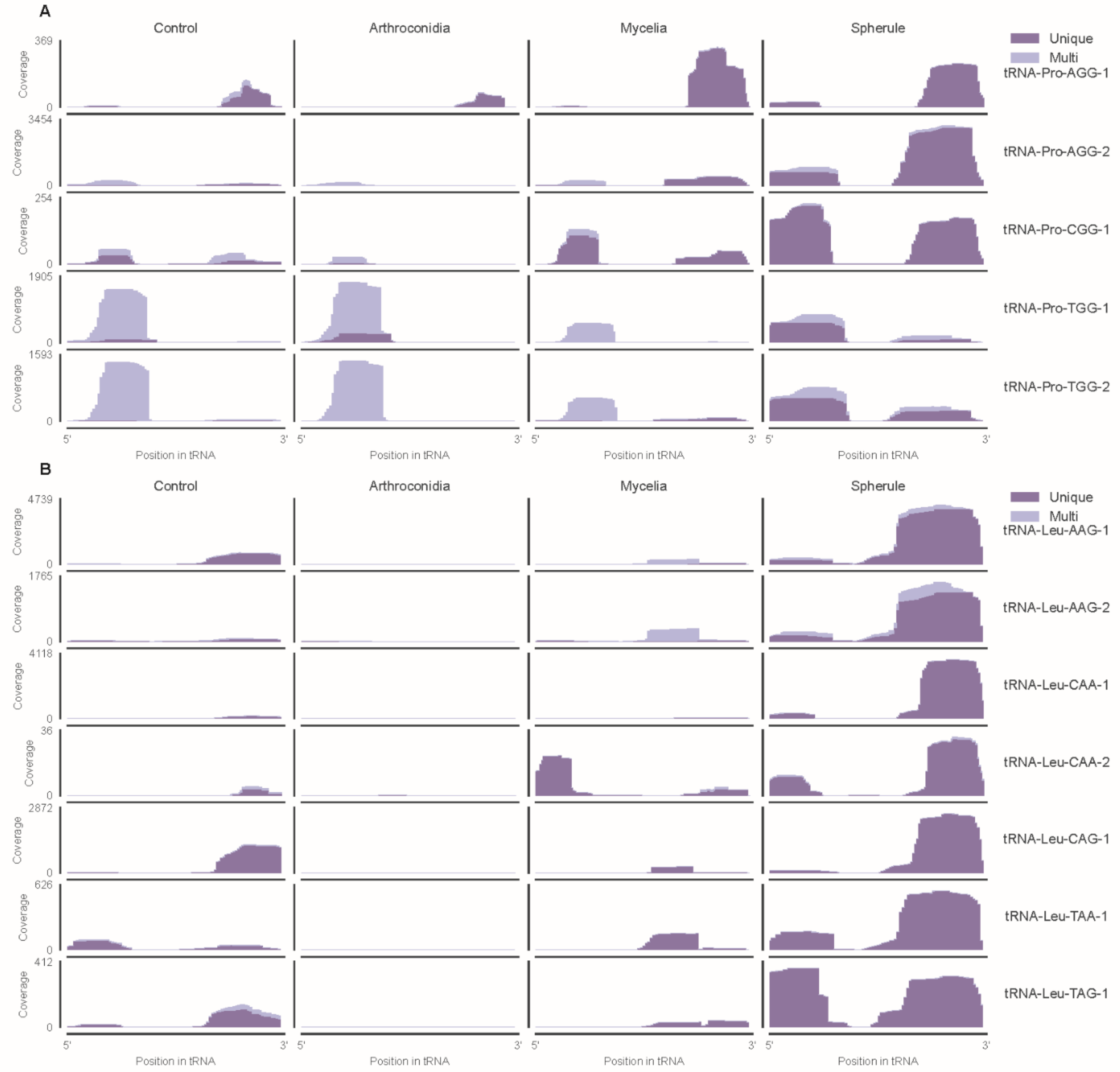


**Supplemental Figure 12**
