# Supplementary material for: Transcriptomic characterization of Coccidioides morphological states using RiboMarker-enhanced RNA sequencing
Source: G3 (Bethesda). 2026 Apr 28;16(7):jkag111. doi: 10.1093/g3journal/jkag111 (PMC13334182; doi:10.1093/g3journal/jkag111)
Supplement: jkag111_Supplementary_Data [file jkag111_supplementary_data.zip › Supplemental_Material_Legends_G3-2026-406762.docx]

**Supporting Information Legends**

**Fig. S1.** RiboMarker®️ Buffer captures the most diverse set of RNA molecules from the tested buffer conditions.

**Fig. S2.** Read length distributions for reads mapping to *C. posadasii* for different morphologies and localizations.

**Fig S3.** Breakdown of RNA transcript annotations for reads mapping to *C. posadasii* for different morphologies and localizations.

**Fig. S4.** Differential sets of RNA transcripts are more readily incorporated into both Biofluids and RiboMarker®️ libraries.

**Fig. S5.** Principal component analysis of intracellular *C. posadasii* samples generated using Biofluids and RiboMarker®️.

**Fig. S6.** Read coverage across transcripts identified as differentially abundant across different morphological stages of *C. posadasii*.

**Fig.** **S7.** Read coverage across tRNA isodecoders that were identified as differentially abundant across different morphological stages of *C. posadasii*. Color is indicative of read mappability with dark purple being uniquely mapped to an individual tRNA isodecoder and light purple mapping to multiple different tRNA isodecoders.

**Fig. S8.** Read coverage of unannotated transcripts identified as differentially abundant across different morphological stages of *C. posadasii*. Predicted secondary structures based on minimum free energy folding are presented for each highlighted area (red boxes; B,D,E) and the entirety of sRNAlocus_4580 (F).

**Fig. S9.** Scatter plot of log2(TPM) values for protein_coding mapped reads from RiboMarker (y-axis) versus those from the Zymo RiboFree Total RNA library preparation (Zymo Research;x-axis). Pearson correlation coefficient values are included for each morphology.

**Fig. S10.** Dot plot representing the number of detected transcript annotations mapped to *C. posadasii* for cell-free (top) and exosome (bottom) samples using RiboMarker®️, with dot size proportional to the log2(abundance) of each RNA class.

**Fig. S11.** Comparative analysis of annotated transcript abundance between exosome and cell-free RNA preparations for arthroconidia, mycelia, and spherule samples from *C. posadasii.* The Pearson correlation coefficient (r) for each comparison is shown at the top of each plot, indicating a strong correlation across all time points and sample types.

**Fig. S12.** Read coverage across tRNA isodecoders that were identified as differentially abundant across exosome fractions of arthroconidia, mycelia, and spherule samples for C. posadasii. Color is indicative of read mappability with purple being uniquely mapped to an individual tRNA isodecoder and blue mapping to multiple different tRNA isodecoders.
